# Correction: Male and female bees show large differences in floral preference

**DOI:** 10.1371/journal.pone.0217714

**Published:** 2019-06-04

**Authors:** The PLOS ONE Staff

There is an error in Fig 2. The image is rotated 90 degrees clockwise from its intended orientation. The publisher apologizes for this error. Please see the complete, correct Fig 2 here.

**Fig 2:**
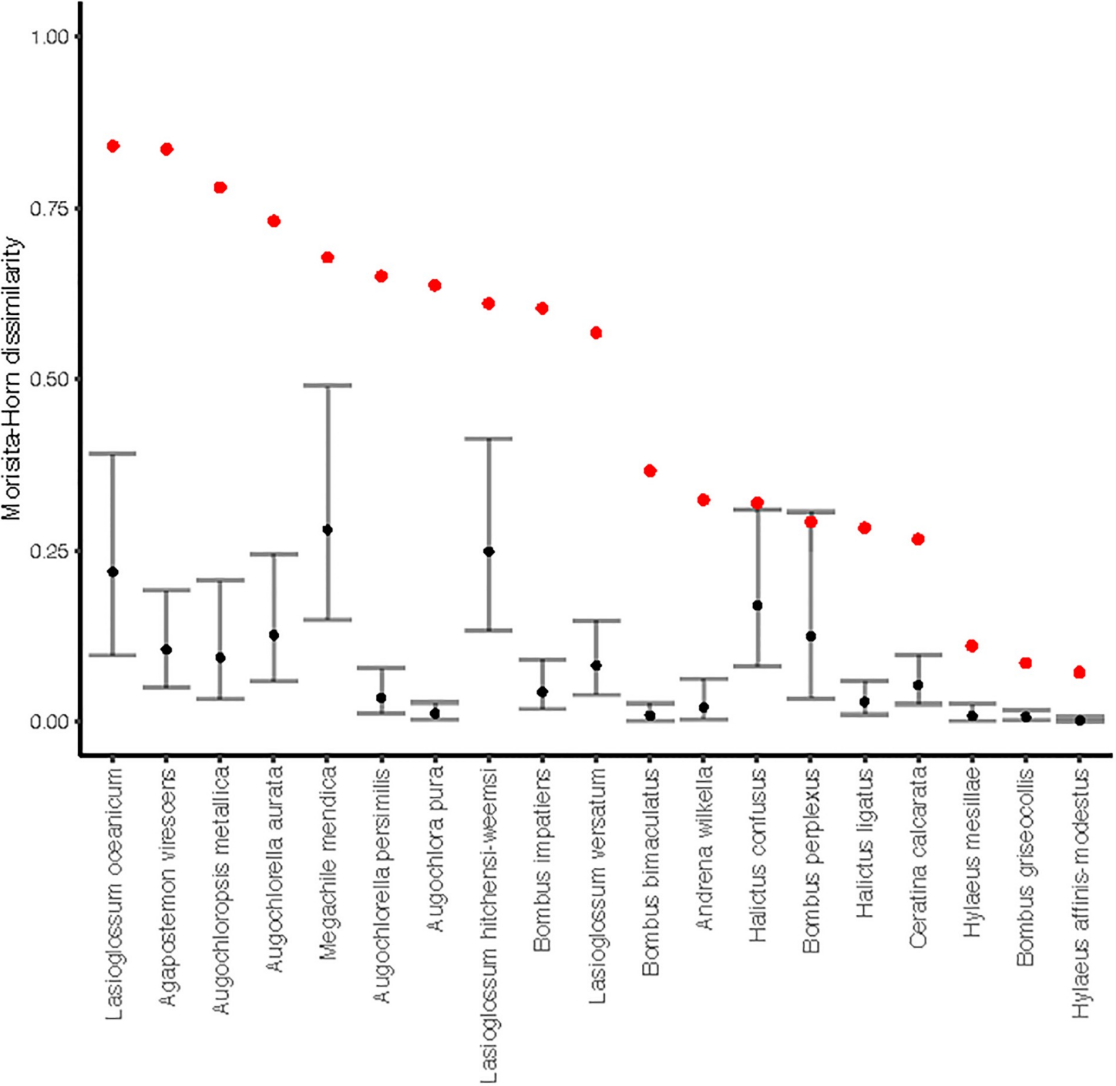
Flower visit patterns of male and female bees of the same species differed significantly. Red points are observed Morisita-Horn dissimilarities between flower communities visited by all male and all female bees of a particular species across all sites and sampling rounds. Black points are the mean dissimilarity (gray bars, 95% CI) from a permutation-based null model that randomly shuffles the sex associated with each visit record, maintaining the total number of males, females, and overall combined visits to each floral species.
