# Supplementary material for: Male and female bees show large differences in floral preference
Source: PLoS One. 2019 Apr 24;14(4):e0214909. doi: 10.1371/journal.pone.0214909 (PMC6481915; doi:10.1371/journal.pone.0214909)
Supplement: S1 File — (a) The six study sites in central New Jersey, USA. (b) Schematic sampling diagram (not to scale). One observer walked parallel 2m transects covering the entire sampling area. Each 30-minute sampling bout resumed where the previous one left off; observers typically covered the entire meadow once over a 3-day sampling round. (c) The southwestern-most site in peak bloom (Figure A). Schematic cartoon of our simulation for the dissimilarity values associated with our null hypothesis that diets of male and female bees do not differ. (a) Each collection record for each bee species associates the sex of an individual bee to the flower species from which it was collected. (b) To compute the dissimilarity between males and females, we compare all visits to each flower species from males (purple vector) to all visits to each flower species from females (green vector). (c) The Morisita-Horn index summarizes the differences between the two vectors as a value between 0 (identical) and 1 (maximally dissimilar). (d) For our null model, we shuffle the sex column from our observation table. (e) This produces two null vectors. The row and column sums for the matrices in (b) and (c) are identical, but the elements can differ. (f) For our null model, we compute the dissimilarity between the null vectors. We repeated steps d-f 9999 times to generate confidence intervals for the null hypothesis that the sex of a visiting bee is unrelated to the flower species it is collected from. When comparing the flower species visited by different species of bee, we conducted an analysis identical except that rather than comparing two sexes of the same species, we compared two species of the same sex (i.e. exchanging “sex” and “species” throughout Figure A in S1 File) (Figure B). Effect size for diet dissimilarity is independent of sample size, while standardized effect is strongly driven by the number of individuals of the sex with the fewest records. a) Observed Morisita-Horn dissimilarity in flowe [file pone.0214909.s001.pdf]

Supporting Information S1

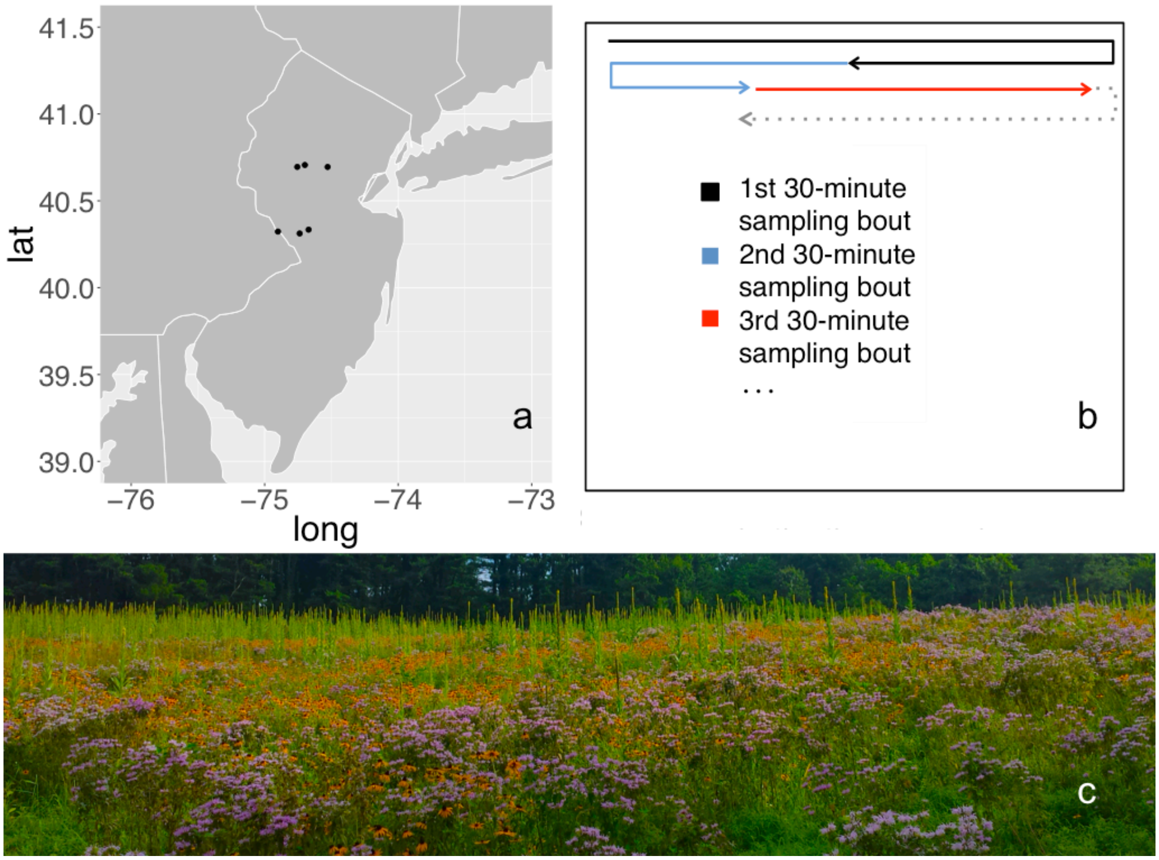

Figure A: Sampling scheme. (a) The six study sites in central New Jersey, USA. (b) Schematic sampling diagram (not to scale). One observer walked parallel 2m transects covering the entire sampling area. Each 30-minute sampling bout resumed where the previous one left off; observers typically covered the entire meadow once over a 3-day sampling round. (c) The southwestern-most site in peak bloom.

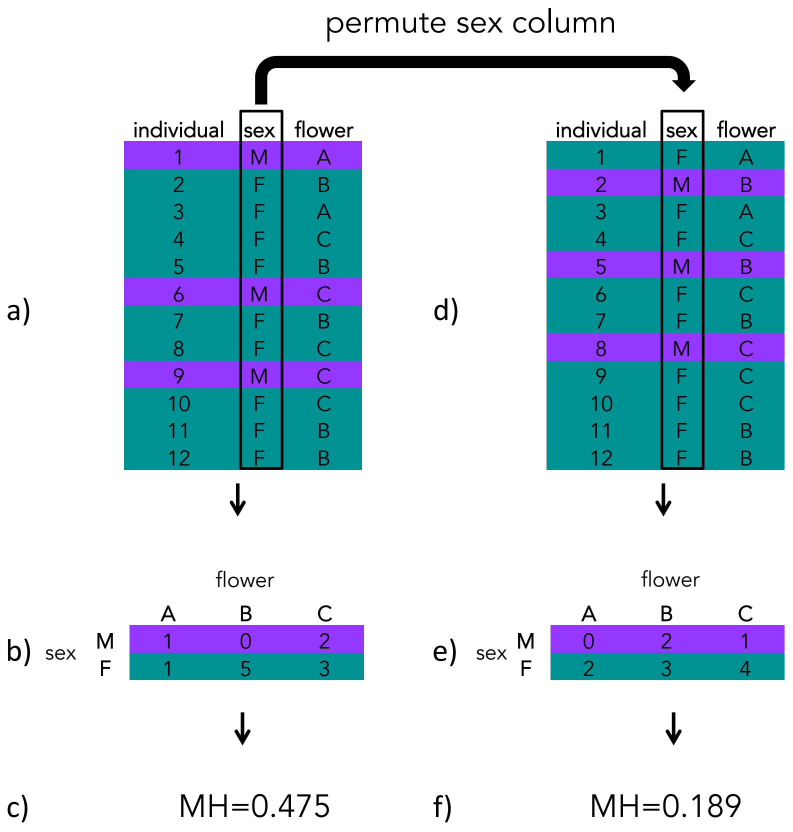

Figure B. Schematic cartoon of our simulation for the dissimilarity values associated with our null hypothesis that diets of male and female bees do not differ. (a) Each collection record for each bee species associates the sex of an individual bee to the flower species from which it was collected. (b) To compute the dissimilarity between males and females, we compare all visits to each flower species from males (purple vector) to all visits to each flower species from females (green vector). (c) The Morisita-Horn index summarizes the differences between the two vectors as a value between 0 (identical) and 1 (maximally dissimilar). (d) For our null model, we shuffle the sex column from our

observation table. (e) This produces two null vectors. The row and column sums for the matrices in (b) and (c) are identical, but the elements can differ. (f) For our null model, we compute the dissimilarity between the null vectors. We repeated steps d-f 9999 times to generate confidence intervals for the null hypothesis that the sex of a visiting bee is unrelated to the flower species it is collected from. When comparing the flower species visited by different species of bee, we conducted an analysis identical except that rather than comparing two sexes of the same species, we compared two species of the same sex (i.e. exchanging "sex" and "species" throughout figure A).

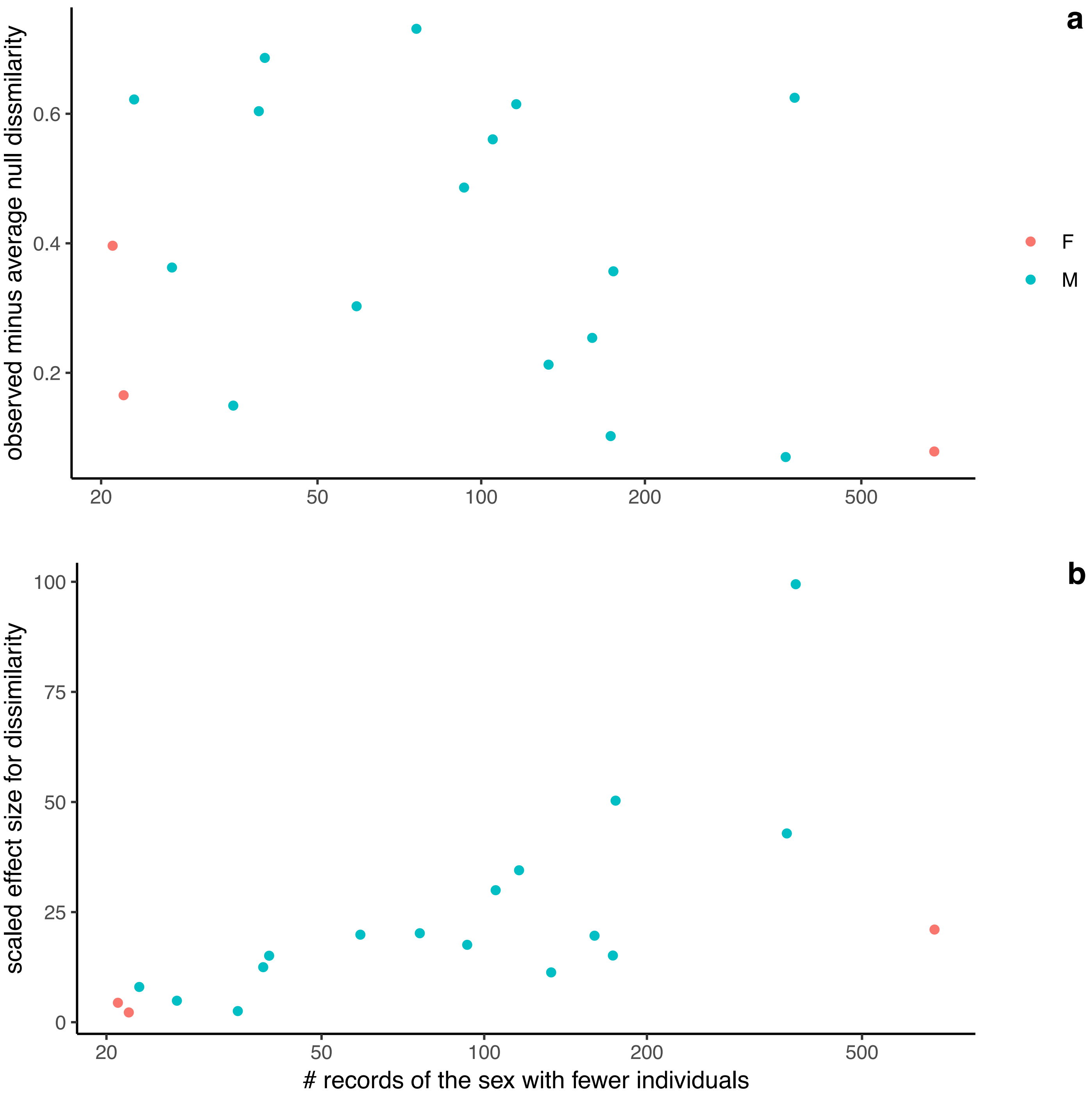

Figure C. Effect size for diet dissimilarity is independent of sample size, while standardized effect is strongly driven by the number of individuals of the sex with the fewest records. a) Observed Morisita-Horn dissimilarity in flower communities visited by male and female bees of a single species, minus average null

dissimilarity vs. the number of records for the less frequently observed sex. b) Observed minus null dissimilarity in composition of flowers visited by male and female bees of a single species, scaled by the variation in the null model, versus the number of records for the less frequently observed sex.

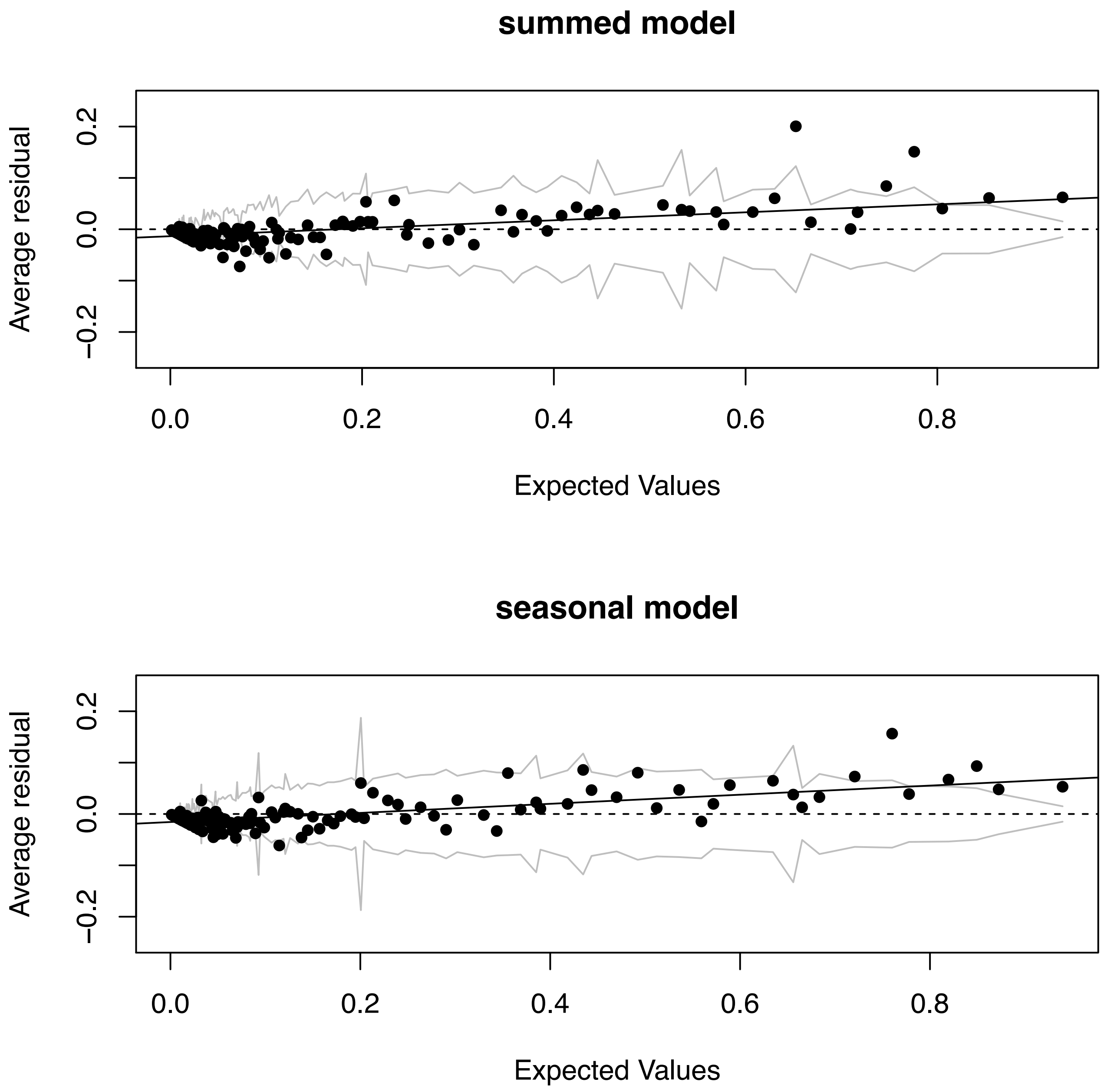

Figure D. Binned residual plots for each model show minor violation of the additivity assumption. Residuals and predicted values on the probability scale.

Appendix A. Methods for post-hoc analysis of male avoidance of nectar-free flowers**.**

Based on previously published work we determined that 11 flower species in our dataset do not produce floral nectar: *Chamaecrista fasciculata* flowers [1]*, Senna hebecarpa* [2]*, Desmodium paniculatum* [3]*, Solanum carolinianum* [4]*, Securigera varia* [4]*, Plantago lanceolata* [5]*, Hypericum perforatum* [6]*, Hypericum punctatum* [6]*, Tradescantia ohioensis* [7], *Sisrynchium angustifolium* [8]*, Glyceria grandis* [4]*, Sorgahastrum nutans* [4].

We compared the mean random effects predictions for each of these species from our seasonal model (main text figure 6) with the random effects predictions for nectar-producing species. We compared the mean value for each set of random effects predictions with a Welch's t-test.The difference was nearly significant according to this test (p=0.055), although the assumption of independence between observations was certainly invalidated by the random effects structure of our model. We report the difference in means as an odds ratio in the text and present boxplots below (Figure S5)

1. Rutter MT, Rausher MD. Natural selection on extrafloral nectar production in Chamaecrista fasciculata: the costs and benefits of a mutualism trait. Evolution (N Y). 2004;58: 2657–2668.
2. Vaudo AD, Patch HM, Mortensen DA, Grozinger CM, Tooker JF. Bumble bees exhibit daily behavioral patterns in pollen foraging. Arthropod Plant

Interact. 2014;8: 273–283. doi:10.1007/s11829-014-9312-5

3. Robertson C. Flowers and Insects IV. Bot Gaz. 1890;15: 79–84.

4. Bernardello G. A systematic survey of floral nectaries. In: Nicolson SW, Nepi M, Pacini E, editors. Nectaries and Nectar. Springer; 2007. pp. 19–128.

5. Sharma N, Koul P, Koul AK. Pollination biology of some species of genus Plantago L. Bot J Linn Soc. 1993;111: 129–138.

6. Willmer P. Pollination and floral ecology. Princeton: Princeton University Press; 2011.

7. Vaudo AD, Patch HM, Mortensen DA, Tooker JF, Grozinger CM. Macronutrient ratios in pollen shape bumble bee (Bombus impatiens) foraging strategies and floral preferences. Proc Natl Acad Sci. 2016;113: E4035–E4042. doi:10.1073/pnas.1606101113

8. Silvério A, Nadot S, Souza-Chies TT, Chauveau O. Floral rewards in the tribe Sisyrinchieae (Iridaceae): Oil as an alternative to pollen and nectar? Sex Plant Reprod. 2012;25: 267–279. doi:10.1007/s00497-012-0196-1

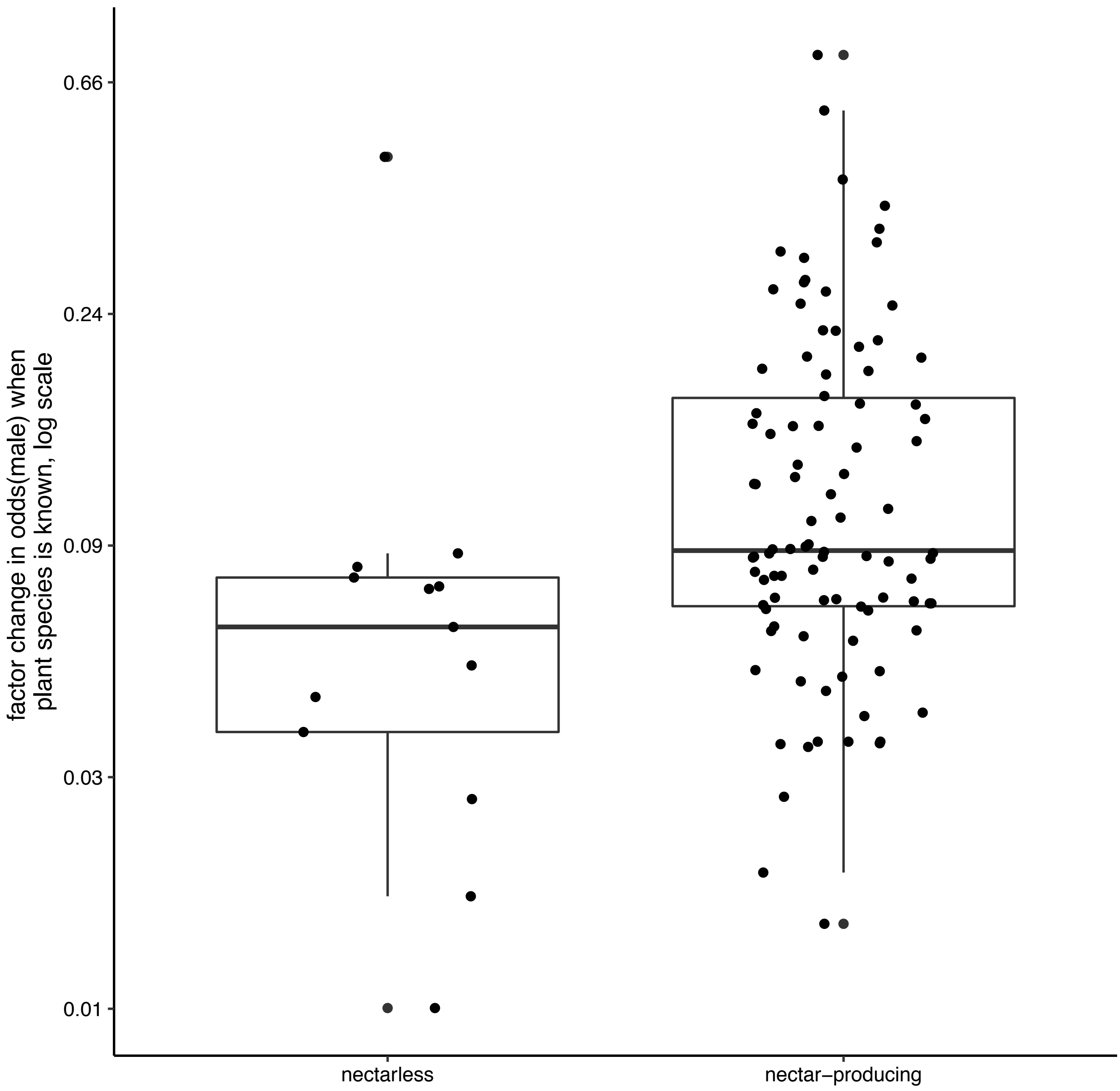

Figure E. Seasonal model predictions are consistent with the hypothesis that male bees avoid flower species that do not produce nectar, relative to females. Each point is the random effect prediction (change in odds that a bee visiting that flower is male) for a flower species. Boxplots show the 25th, 50th, and 75th

percentiles, with whiskers extending to more extreme values within 1.5x the interquartile range.

95 **Table A.** Model convergence confirmed based on similar parameter estimates across fitting routines. For each model, the estimate for each term is given for each of 6 fitting algorithms in the R package lme4. Subsequent analyses used parameter estimates in yellow, in both cases tied for the highest estimated likelihood with other very similar fits.

**term**	**model**	bobyqa	Nelder_Mead	nlminbw	optimx.L-BFGS-B	nloptwrap.NLOPT_LN_NELDERMEAD	nloptwrap.NLOPT_LN_BOBYQA
intercept	summed	-2.43	-2.43	-2.43	-2.43	-2.43	-2.43
bee species	summed	2.04	2.04	2.04	2.04	2.04	2.04
flower species	summed	1.40	1.40	1.40	1.40	1.40	1.40
site	summed	0.00	0.00	0.00	0.00	0.00	0.00
bee species:flower species	summed	1.21	1.21	1.21	1.21	1.21	1.21
site:bee species	summed	0.62	0.62	0.62	0.62	0.62	0.62
site:flower species	summed	0.61	0.61	0.61	0.61	0.61	0.61
intercept	seasonal	-2.38	-2.45	-2.38	-2.38	-2.45	-2.45
bee species	seasonal	2.09	2.14	2.09	2.09	2.13	2.13
flower species	seasonal	1.25	1.27	1.25	1.25	1.27	1.27

site	seasonal	0.00	0.00	0.00	0.00	0.00	0.00
bee species:flower species	seasonal	1.09	1.10	1.09	1.09	1.10	1.10
site:bee species	seasonal	0.46	0.47	0.46	0.46	0.47	0.47
site:flower species	seasonal	0.35	0.35	0.35	0.35	0.35	0.35
sampling round	seasonal	0.38	0.36	0.38	0.38	0.36	0.36
sampling round:bee species	seasonal	0.83	0.84	0.83	0.83	0.84	0.84
sampling round:flower species	seasonal	0.00	0.00	0.00	0.00	0.00	0.00
sampling round:site	seasonal	0.29	0.29	0.29	0.29	0.29	0.29
sampling round:site:bee species	seasonal	0.60	0.60	0.60	0.60	0.60	0.60
sampling round:site:flower species	seasonal	0.28	0.29	0.28	0.28	0.28	0.28

Table B. Bee species with number of female and male specimens collected.

family	genus	species	females	males
Andrenidae	*Andrena*	*brevipalpis*	1	0
Andrenidae	*Andrena*	*carlini*	3	0
Andrenidae	*Andrena*	*commoda*	3	0
Andrenidae	*Andrena*	*cressonii*	16	0
Andrenidae	*Andrena*	*fragilis*	2	0
Andrenidae	*Andrena*	*hippotes*	4	0
Andrenidae	*Andrena*	*imitatrix*	6	0
Andrenidae	*Andrena*	*krigiana*	14	0
Andrenidae	*Andrena*	*nasonii*	13	0
Andrenidae	*Andrena*	*nuda*	2	0
Andrenidae	*Andrena*	*pruni*	6	0
Andrenidae	*Andrena*	*robertsonii*	8	0
Andrenidae	*Andrena*	*rudbeckiae*	8	11
Andrenidae	*Andrena*	*rugosa*	1	0
Andrenidae	*Andrena*	*spiraeana*	1	0
Andrenidae	*Andrena*	*vicina*	6	0
Andrenidae	*Andrena*	*wilkella*	277	59
Andrenidae	*Andrena*	*wilmattae*	2	0
Andrenidae	*Calliopsis*	*andreniformis*	4	1
Apidae	*Anthophora*	*abrupta*	4	0
Apidae	*Anthophora*	*terminalis*	3	2
Apidae	*Bombus*	*auricomus*	1	0
Apidae	*Bombus*	*bimaculatus*	577	175
Apidae	*Bombus*	*citrinus*	0	5
Apidae	*Bombus*	*fervidus*	18	0
Apidae	*Bombus*	*griseocollis*	681	815
Apidae	*Bombus*	*impatiens*	2358	105
Apidae	*Bombus*	*perplexus*	22	36
Apidae	*Bombus*	*vagans*	14	2
Apidae	*Ceratina*	*calcarata*	1417	133
Apidae	*Ceratina*	*dupla*	151	19
Apidae	*Ceratina*	*mikmaqi*	130	5
Apidae	*Ceratina*	*strenua*	285	13

Apidae	*Melissodes*	*agilis*	0	7
Apidae	*Melissodes*	*bimaculatus*	9	1
Apidae	*Melissodes*	*denticulatus*	7	73
Apidae	*Melissodes*	*desponsus*	1	7
Apidae	*Melissodes*	*subillatus*	31	6
Apidae	*Melissodes*	*trinodis*	1	7
Apidae	*Nomada*	*articulata*	4	0
Apidae	*Nomada*	*bidentate_gr*	8	0
Apidae	*Nomada*	*erigeronis*	1	0
Apidae	*Nomada*	*lehighensis*	1	0
Apidae	*Nomada*	*maculata*	2	0
Apidae	*Nomada*	*pygmaea*	15	0
Apidae	*Ptilothrix*	*bombiformis*	0	1
Apidae	*Triepeolus*	*cressonii*	0	1
Apidae	*Triepeolus*	*eliseae*	1	0
Apidae	*Triepeolus*	*remigatus*	1	0
Apidae	*Xylocopa*	*virginica*	137	13
Colletidae	*Hylaeus*	*affinis_modestus*	1376	363
Colletidae	*Hylaeus*	*fedorica*	1	0
Colletidae	*Hylaeus*	*leptocephalus*	1	3
Colletidae	*Hylaeus*	*mesillae*	575	173
Halictidae	*Agapostemon*	*sericeus*	5	5
Halictidae	*Agapostemon*	*virescens*	203	76
Halictidae	*Augochlora*	*pura*	1036	377
Halictidae	*Augochlorella*	*aurata*	397	39
Halictidae	*Augochlorella*	*persimilis*	434	116
Halictidae	*Augochloropsis*	*metallica*	121	40
Halictidae	*Dufourea*	*novaeangliae*	0	1
Halictidae	*Halictus*	*confusus*	174	35
Halictidae	*Halictus*	*ligatus*	2432	160
Halictidae	*Halictus*	*parallelus*	6	18
Halictidae	*Halictus*	*rubicundus*	31	19
Halictidae	*Lasioglossum*	*abanci*	6	0
Halictidae	*Lasioglossum*	*admirandum*	15	0
Halictidae	*Lasioglossum*	*anomalum*	17	0
Halictidae	*Lasioglossum*	*atwoodi*	7	1
Halictidae	*Lasioglossum*	*birkmanni*	1	0
Halictidae	*Lasioglossum*	*bruneri*	6	4
Halictidae	*Lasioglossum*	*callidum*	54	0
Halictidae	*Lasioglossum*	*cattellae*	14	4
Halictidae	*Lasioglossum*	*coeruleum*	2	0

Halictidae	*Lasioglossum*	*coreopsis*	1	0
Halictidae	*Lasioglossum*	*coriaceum*	14	0
Halictidae	*Lasioglossum*	*cressonii*	16	5
Halictidae	*Lasioglossum*	*ellisiae*	0	3
Halictidae	*Lasioglossum*	*ephialtum*	1	0
Halictidae	*Lasioglossum*	*foxii*	2	2
Halictidae	*Lasioglossum*	*fuscipenne*	9	0
Halictidae	*Lasioglossum*	*gotham*	74	2
Halictidae	*Lasioglossum*	*hitchensi_weemsi*	152	27
Halictidae	*Lasioglossum*	*illinoense*	70	7
Halictidae	*Lasioglossum*	*imitatum*	462	15
Halictidae	*Lasioglossum*	*leucocomum*	2	0
Halictidae	*Lasioglossum*	*leucozonium*	2	0
Halictidae	*Lasioglossum*	*nigroviride*	2	0
Halictidae	*Lasioglossum*	*oblongum*	4	2
Halictidae	*Lasioglossum*	*obscurum*	7	1
Halictidae	*Lasioglossum*	*oceanicum*	104	23
Halictidae	*Lasioglossum*	*oenotherae*	1	0
Halictidae	*Lasioglossum*	*paradmirandum*	50	0
Halictidae	*Lasioglossum*	*pectorale*	3	0
Halictidae	*Lasioglossum*	*pilosum*	2	0
Halictidae	*Lasioglossum*	*platyparium*	2	3
Halictidae	*Lasioglossum*	*rozeni*	15	11
Halictidae	*Lasioglossum*	*smilacinae*	4	0
Halictidae	*Lasioglossum*	*subviridatum*	5	1
Halictidae	*Lasioglossum*	*tegulare*	31	2
Halictidae	*Lasioglossum*	*trigeminum*	44	0
Halictidae	*Lasioglossum*	*truncatum*	2	0
Halictidae	*Lasioglossum*	*versatum*	681	93
Halictidae	*Lasioglossum*	*viridatum*	11	2
Halictidae	*Lasioglossum*	*zephyrum*	12	1
Halictidae	*Sphecodes*	*atlantis*	0	1
Halictidae	*Sphecodes*	*dichrous*	3	5
Halictidae	*Sphecodes*	*heraclei*	10	5
Megachilidae	*Anthidiellum*	*notatum*	4	1
Megachilidae	*Anthidium*	*manicatum*	7	8
Megachilidae	*Anthidium*	*oblongatum*	18	19
Megachilidae	*Coelioxys*	*alternatus*	1	2
Megachilidae	*Coelioxys*	*banksi*	1	0
Megachilidae	*Coelioxys*	*germanus*	0	1
Megachilidae	*Coelioxys*	*hunteri*	0	1

Megachilidae	*Coelioxys*	*modestus*	0	1
Megachilidae	*Coelioxys*	*obtusiventris*	1	0
Megachilidae	*Coelioxys*	*octodentatus*	1	1
Megachilidae	*Coelioxys*	*porterae*	0	1
Megachilidae	*Coelioxys*	*sayi*	2	6
Megachilidae	*Heriades*	*carinatus*	31	2
Megachilidae	*Heriades*	*leavitti*	1	6
Megachilidae	*Heriades*	*variolosus*	10	0
Megachilidae	*Hoplitis*	*pilosifrons*	46	1
Megachilidae	*Hoplitis*	*producta*	8	0
Megachilidae	*Hoplitis*	*spoliata*	2	1
Megachilidae	*Lithurgus*	*chrysurus*	0	6
Megachilidae	*Megachile*	*brevis*	25	3
Megachilidae	*Megachile*	*campanulae*	6	18
Megachilidae	*Megachile*	*exilis*	11	29
Megachilidae	*Megachile*	*frugalis*	26	6
Megachilidae	*Megachile*	*gemula*	4	2
Megachilidae	*Megachile*	*georgica*	1	0
Megachilidae	*Megachile*	*inimica*	4	0
Megachilidae	*Megachile*	*integra*	1	0
Megachilidae	*Megachile*	*melanophaea*	0	1
Megachilidae	*Megachile*	*mendica*	22	56
Megachilidae	*Megachile*	*montivaga*	15	9
Megachilidae	*Megachile*	*petulans*	0	2
Megachilidae	*Megachile*	*pugnata*	2	3
Megachilidae	*Megachile*	*rotundata*	11	8
Megachilidae	*Megachile*	*sculpturalis*	17	32
Megachilidae	*Megachile*	*xylocopoides*	2	1
Megachilidae	*Osmia*	*albiventris*	3	0
Megachilidae	*Osmia*	*atriventris*	9	0
Megachilidae	*Osmia*	*bucephala*	21	0
Megachilidae	*Osmia*	*distincta*	7	0
Megachilidae	*Osmia*	*georgica*	5	0
Megachilidae	*Osmia*	*pumila*	30	0
Megachilidae	*Pseudoanthidium*	*nanum*	0	1
Megachilidae	*Stelis*	*lateralis*	1	0
Megachilidae	*Stelis*	*louisae*	1	2

Table C. Number of male and female visitors to each plant species, and bias towards attracting male bee visitors. This bias is the random effect prediction

from the seasonal model, which indicates the change in log(odds) that a visiting bee is male when the species of flower it visits is given; greater values indicate male bias.

family	genus	species	female visits	male visits	random effect
Verbenaceae	*Verbena*	*urticifolia*	58	71	2.117
Asteraceae	*Erechtites*	*hieraciifolius*	130	203	1.880
Fabaceae	*Senna*	*hebecarpa*	5	5	1.680
Phytolaccaceae	*Phytolacca*	*americana*	108	74	1.581
Asteraceae	*Euthamia*	*graminifolia*	50	18	1.468
Asteraceae	*Eutrochium*	*maculatum*	461	166	1.367
Fabaceae	*Melilotus*	*officinalis*	41	21	1.308
Lamiaceae	*Nepeta*	*cataria*	99	81	1.270
Lamiaceae	*Monarda*	*punctata*	0	1	1.243
Campanulaceae	*Lobelia*	*inflata*	12	3	1.146
Asteraceae	*Liatris*	*spicata*	186	128	1.140
Asteraceae	*Solidago*	*juncea*	636	77	1.104
Asteraceae	*Conyza*	*canadensis*	32	10	1.097
Polygonaceae	*Fallopia*	*convolvulus*	3	4	1.046
Asteraceae	*Erigeron*	*strigosus*	712	119	1.036
Asclepidaceae	*Asclepias*	*syriaca*	28	89	0.929
Verbenaceae	*Verbena*	*hastata*	8	3	0.925
Lamiaceae	*Pycnanthemum*	*verticillatum*	5	8	0.886
Asteraceae	*Cirsium*	*arvense*	351	96	0.859
Lamiaceae	*Pycnanthemum*	*muticum*	398	60	0.817
Asteraceae	*Solidago*	*canadensis*	8	2	0.816
Asteraceae	*Heliopsis*	*helianthoides*	186	49	0.763
Apocynaceae	*Apocynum*	*cannabinum*	283	92	0.754
Fabaceae	*Trifolium*	*hybridum*	11	7	0.742
Asteraceae	*Rudbeckia*	*hirta*	1174	189	0.645
Asteraceae	*Solidago*	*gigantea*	107	9	0.613
Lamiaceae	*Pycnanthemum*	*tenuifolium*	1113	421	0.608
Rosaceae	*Drymocallis*	*arguta*	22	8	0.604
Fabaceae	*Melilotus*	*albus*	20	9	0.570
Cornaceae	*Swida*	*racemosa*	12	1	0.544
Apiaceae	*Daucus*	*carota*	1783	350	0.523
Asteraceae	*Helianthus*	*strumosus*	1	1	0.518
Asteraceae	*Cichorium*	*intybus*	104	10	0.513
Verbenaceae	*Verbena*	*simplex*	11	3	0.482

Asteraceae	*Symphyotrichum*	*novae-angliae*	25	6	0.451
Asclepidaceae	*Asclepias*	*tuberosa*	114	26	0.427
Asteraceae	*Cirsium*	*discolor*	5	2	0.350
Asteraceae	*Echinacea*	*purpurea*	86	45	0.314
Lamiaceae	*Prunella*	*vulgaris*	17	5	0.295
Asteraceae	*Centuarea*	*stoebe*	321	50	0.268
Asteraceae	*Erigeron*	*annuus*	26	3	0.264
Onagraceae	*Oenothera*	*fruticosa*	2	1	0.220
Polygonaceae	*Persicaria*	*setacea*	3	1	0.158
Asteraceae	*Cirsium*	*vulgare*	112	16	0.121
Fabaceae	*Trifolium*	*campestre*	365	39	0.110
Asteraceae	*Ratibida*	*pinnata*	539	121	0.012
Asteraceae	*Achillea*	*millefolium*	473	36	-0.008
Asteraceae	*Bidens*	*trichosperma*	1	0	-0.015
Asteraceae	*Solidago*	*rugosa*	1	0	-0.015
Lythraceae	*Lythrum*	*salicaria*	364	38	-0.017
Rosaceae	*Rubus*	*flagellaris*	1	0	-0.028
Fabaceae	*Trifolium*	*aureum*	1	0	-0.033
Campanulaceae	*Lobelia*	*siphilitica*	2	0	-0.034
Gentianaceae	*Sabatia*	*angularis*	1	0	-0.034
Asteraceae	*Vernonia*	*noveboracensis*	52	37	-0.044
Fabaceae	*Vicia*	*tetrasperma*	1	0	-0.048
Apiaceae	*Sanicula*	*canadensis*	1	0	-0.049
Asteraceae	*Doellingeria*	*umbellata*	1	0	-0.052
Ranunculaceae	*Ranunculus*	*hispidus*	1	0	-0.057
Asteraceae	*Coreopsis*	*tinctoria*	1	0	-0.068
Poaceae	*Sorghastrum*	*nutans*	1	0	-0.092
Lamiaceae	*Teucrium*	*canadense*	3	0	-0.104
Brassicaceae	*Barbarea*	*vulgaris*	3	0	-0.115
Loniceraceae	*Lonicera*	*japonica*	1	0	-0.129
Lamiaceae	*Monarda*	*fistulosa*	1398	401	-0.132
Fabaceae	*Desmodium*	*paniculatum*	6	1	-0.137
Onagraceae	*Oenothera*	*biennis*	2	0	-0.143
Asteraceae	*Hieracium*	*pilosella*	2	0	-0.149
Hypericaceae	*Hypericum*	*punctatum*	1	0	-0.177
Poaceae	*Glyceria*	*grandis*	1	0	-0.187
Alliaceae	*Allium*	*vineale*	3	0	-0.225
Apiaceae	*Eryngium*	*yuccifolium*	2	0	-0.226
Fabaceae	*Lotus*	*corniculatus*	142	33	-0.228
Asteraceae	*Crepis*	*capillaris*	6	0	-0.236
Rosaceae	*Rubus*	*pensilvanicus*	7	0	-0.242

Cornaceae	*Swida*	*amomum*	4	0	-0.250
Oxalidaceae	*Oxalis*	*stricta*	8	0	-0.250
Asteraceae	*Lactuca*	*serriola*	4	0	-0.257
Rosaceae	*Rosa*	*multiflora*	5	0	-0.264
Asteraceae	*Gaillardia*	*aristata*	4	0	-0.275
Caryophyllaceae	*Dianthus*	*armeria*	3	0	-0.281
Iridaceae	*Sisyrinchium*	*angustifolium*	9	0	-0.353
Asteraceae	*Carduus*	*nutans*	1	0	-0.367
Rubiaceae	*Galium*	*mollugo*	4	0	-0.369
Polygonaceae	*Persicaria*	*pensylvanica*	4	0	-0.392
Asteraceae	*Leucanthemum*	*vulgare*	406	20	-0.397
Asteraceae	*Helianthus*	*angustifolius*	11	0	-0.412
Solanaceae	*Solanum*	*carolinense*	14	0	-0.517
Convulvulaceae	*Calystegia*	*silvatica*	5	0	-0.539
Asteraceae	*Krigia*	*biflora*	19	0	-0.544
Fabaceae	*Baptisia*	*tinctoria*	19	5	-0.566
Asteraceae	*Solidago*	*altissima*	8	0	-0.587
Rosaceae	*Potentilla*	*recta*	56	1	-0.629
Fabaceae	*Securigera*	*varia*	38	1	-0.653
Scrophulariaceae	*Verbascum*	*blattaria*	15	0	-0.722
Asclepidaceae	*Asclepias*	*incarnata*	7	0	-0.737
Commelinaceae	*Tradescantia*	*ohiensis*	34	1	-0.804
Scrophulariaceae	*Penstemon*	*hirsutus*	36	0	-0.823
Scrophulariaceae	*Penstemon*	*digitalis*	862	48	-0.842
Fabaceae	*Trifolium*	*repens*	130	6	-0.848
Lamiaceae	*Clinopodium*	*vulgare*	64	3	-0.855
Asteraceae	*Coreopsis*	*lanceolata*	21	0	-0.857
Fabaceae	*Trifolium*	*pratense*	192	20	-0.869
Scrophulariaceae	*Verbascum*	*thapsus*	129	1	-1.085
Hypericaceae	*Hypericum*	*perforatum*	223	10	-1.087
Rosaceae	*Rosa*	*carolina*	71	1	-1.413
Fabaceae	*Chamaecrista*	*fasciculata*	246	2	-1.514
Scrophulariaceae	*Linaria*	*vulgaris*	275	4	-1.635
Plantaginaceae	*Plantago*	*lanceolata*	147	0	-1.999

109
